# Single‐Cell RNA‐Sequencing Reveals the Breadth of Osteoblast Heterogeneity

**DOI:** 10.1002/jbm4.10496

**Published:** 2021-05-17

**Authors:** Hirotaka Yoshioka, Saki Okita, Masashi Nakano, Tomoko Minamizaki, Asako Nubukiyo, Yusuke Sotomaru, Edith Bonnelye, Katsuyuki Kozai, Kotaro Tanimoto, Jane E Aubin, Yuji Yoshiko

**Affiliations:** ^1^ Department of Calcified Tissue Biology, Graduate School of Biomedical and Health Sciences Hiroshima University Hiroshima Japan; ^2^ Department of Anatomy School of Medicine, International University of Health and Welfare Chiba Japan; ^3^ Department of Craniofacial and Developmental Biology, Graduate School of Biomedical and Health Sciences Hiroshima University Hiroshima Japan; ^4^ Department of Pediatric Dentistry, Graduate School of Biomedical and Health Sciences Hiroshima University Hiroshima Japan; ^5^ Department of Pediatric Dentistry Hiroshima University Hospital Hiroshima Japan; ^6^ Natural Science Center of Basic Research and Development Hiroshima University Hiroshima Japan; ^7^ CNRS ERL 6001/INSERM U1232 Institut de Cancérologie de l'Ouest Saint‐Herblain France; ^8^ Department of Molecular Genetics University of Toronto Toronto Canada

**Keywords:** HETEROGENEITY, OSTEOBLAST, RNA SEQUENCING, SINGLE‐CELL

## Abstract

The current paradigm of osteoblast fate is that the majority undergo apoptosis, while some further differentiate into osteocytes and others flatten and cover bone surfaces as bone lining cells. Osteoblasts have been described to exhibit heterogeneous expression of a variety of osteoblast markers at both transcriptional and protein levels. To explore further this heterogeneity and its biological significance, Venus‐positive (Venus^+^) cells expressing the fluorescent protein Venus under the control of the 2.3‐kb *Col1a1* promoter were isolated from newborn mouse calvariae and subjected to single‐cell RNA sequencing. Functional annotation of the genes expressed in 272 Venus^+^ single cells indicated that Venus^+^ cells are osteoblasts that can be categorized into four clusters. Of these, three clusters (clusters 1 to 3) exhibited similarities in their expression of osteoblast markers, while one (cluster 4) was distinctly different. We identified a total of 1920 cluster‐specific genes and pseudotime ordering analyses based on established concepts and known markers showed that clusters 1 to 3 captured osteoblasts at different maturational stages. Analysis of gene co‐expression networks showed that genes involved in protein synthesis and protein trafficking between endoplasmic reticulum (ER) and Golgi are active in these clusters. However, the cells in these clusters were also defined by extensive heterogeneity of gene expression, independently of maturational stage. Cells of cluster 4 expressed *Cd34* and *Cxcl12* with relatively lower levels of osteoblast markers, suggesting that this cell type differs from actively bone‐forming osteoblasts and retain or reacquire progenitor properties. Based on expression and machine learning analyses of the transcriptomes of individual osteoblasts, we also identified genes that may be useful as new markers of osteoblast maturational stages. Taken together, our data show much more extensive heterogeneity of osteoblasts than previously documented, with gene profiles supporting diversity of osteoblast functional activities and developmental fates. © 2021 The Authors. *JBMR Plus* published by Wiley Periodicals LLC on behalf of American Society for Bone and Mineral Research.

## Introduction

Bone marrow stromal cells (BMSCs; aka mesenchymal stem cells) have capacity to differentiate into multiple cell types including osteoblasts, adipocytes, and chondrocytes.^(^ ^1^, ^2^ ^)^ Osteoblast lineage fate decision is driven by the master transcription factor RUNX2,^(^ ^3^ ^)^ which directly regulates the expression of SP7, a transcriptional activator for osteoblast differentiation, resulting in recruitment of SP7 and co‐factor DLX to osteoblast enhancers to promote the expression of osteoblast‐specific genes.^(^ ^4^, ^5^ ^)^ Osteoblasts play a pivotal role in bone formation by producing and secreting bone matrix components and initiating matrix mineralization. More than half of the osteoblasts undergo apoptosis, while the remaining cells are entrapped in the bone matrix and become osteocytes or cover inactive (non‐remodeling) bone surfaces as bone lining cells.^(^ ^6^ ^)^ Osteoblasts survive for several weeks, while osteocytes build cellular networks and can survive for more than 20 years.^(^ ^7^ ^)^ Bone lining cells are post‐mitotic flattened cells, which can be reprogrammed to active osteoblasts during adulthood^(^ ^8^ ^)^ in response to external stimuli.^(^ ^9^ ^)^ Thus, lineage commitment and differentiation into osteoblasts are usually considered unidirectional deterministic processes, characterized by at least three different osteogenic fates or outcomes. However, growing evidence shows that fate shifts of osteoblast lineage cells can occur.^(^ ^10^, ^11^ ^)^ For example, a subset of relatively mature rat osteoblasts expressing PPARγ become adipocytes when cultured with the synthetic PPARγ agonist rosiglitazone.^(^ ^10^ ^)^ Loss of Wnt/β‐catenin signaling also changes the fate of preosteoblasts to adipocytes.^(^ ^11^ ^)^ These data suggest that osteoblast lineage cells do not always undergo unidirectional differentiation, but the molecular mechanisms by which osteoblasts may acquire diverse fates remain to be more fully explored.

Single‐cell colony assays^(^ ^12^ ^)^ and in situ hybridization and immunohistochemical analyses^(^ ^13^ ^)^ of osteoblast marker genes have suggested that osteoblasts comprise molecularly heterogeneous populations, which may reflect not only molecular diversity but also functional diversity in osteoblasts.^(^ ^7^, ^12^, ^13^ ^)^ Among the many facets of cellular heterogeneity, non‐genetic (phenotypic) heterogeneity is increasingly being appreciated as not just noise or technical artifact but as a fundamental intrinsic condition not only for the evolution of organismal robustness but also for the relationship between genetic and developmental robustness, including multipotency and cell‐type diversification.^(^ ^14^, ^15^ ^)^ Until recently, analytic tools for transcriptomics were reliably applied mainly to bulk cell samples, but newer technological breakthroughs now allow for transcriptomic analysis at the single‐cell level.^(^ ^16^, ^17^ ^)^ In this study, we sought to demonstrate heterogeneity in osteoblasts isolated from calvariae of newborn mice expressing the fluorescent protein Venus under the control of the 2.3‐kb *Col1a1* promoter (Venus^+^ osteoblasts) by single‐cell transcriptome analysis.

## Materials and Methods

### Generation of *Col1a1‐Cre*; *R26R‐Lyn‐Venus* reporter mice

Transgenic mice expressing Cre recombinase under the control of the 2.3 kb type I collagen promoter (*Col1a1‐Cre*) were obtained from the RIKEN BioResource Center.^(^ ^18^ ^)^ *Col1a1‐Cre* mice were mated with *R26R‐Lyn‐Venus* mice (kindly provided by RIKEN Center for Life Science Technologies; CDB0219K, http://www2.clst.riken.jp/arg/reporter_mice.html)^(^ ^19^ ^)^ to obtain conditional reporter mice expressing the yellow fluorescence protein Venus in osteoblasts (*Col1a1‐Cre*; *R26R‐Lyn‐Venus*). All mice were fed *ad libitum* with a regular diet. Animal use and procedures were approved by the Committee of Animal Experimentation at Hiroshima University.

### Immunohistochemistry

To confirm the distribution of Venus^+^ cells, newborn calvariae were dissected away from surrounding tissue and fixed in 2% paraformaldehyde, 75 mM L‐lysine, 10 mM sodium periodate in 0.1 M phosphate buffer, pH 7.4 at 4°C for 2 hours, demineralized in 10% EDTA in PBS at 4°C for 24 hours, and embedded in paraffin. Deparaffinized sections were pretreated with antigen retrieval solution (6 M urea in 0.1 M Tris–HCl, pH 10.2) for 1 hour at room temperature. Tissue sections (4 to 5 μm thickness) were treated with Protein Block (DAKO, Glostrup, Denmark) for 10 minutes at room temperature, followed by incubation with primary antibodies or negative control IgGs at 4°C overnight. Primary antibodies were against alkaline phosphatase (ALP, 1:100; Proteintech, Chicago, IL, USA) or GFP (Venus, 1:100; Thermo Fisher Scientific, Carlsbad, CA, USA). Goat anti‐rabbit IgG, Alexa Fluor 594 (1:500; Thermo Fisher Scientific) and goat anti‐chicken IgY, Alexa Fluor 488 (1500; Abcam, Cambridge, MA, USA) were used as secondary antibodies. Each incubation step was followed by three washes with TBS including 0.025% Triton X‐100. Fluorokeeper with DAPI (Nacalai Tesque, Tokyo, Japan) was used for counterstaining, and signals were observed under an inverted fluorescence microscope (Leica DMi8; Leica Microsystems, Buffalo Grove, IL, USA).

### Isolation of calvaria cells

Calvaria cells were harvested from 2‐ to 4‐day‐old *Col1a1‐Cre*; *R26R‐Lyn‐Venus* newborn mice as described on the website https://www.csr-mgh.org (The Center for Skeletal Research, Massachusetts General Hospital Endocrine Unit). Briefly, calvariae were aseptically dissected and subjected to 8 sequential digestions (the 1st to 4th, 6th, and 8th steps with 1 mg/mL collagenase type I and II (ratio 1:3; Worthington Biochemical, Lakewood, NJ, USA) in α‐MEM supplemented with 0.1% bovine serum albumin, 15 mM HEPES pH 7.4, 1 mM CaCl_2_; the 5th and 7th steps with 5 mM EDTA in PBS including 0.1% bovine serum albumin). Cells were isolated from each step (fractions 1 to 8); of these, we used fractions 3 to 6 to obtain osteoblasts (see below) and eliminate non‐osteoblastic (see Results) and osteocyte contamination (https://www.csr-mgh.org).^(^ ^20^, ^21^ ^)^

### Cell cultures and cytochemistry

To evaluate their manifestation of the osteoblast phenotype in vitro, Venus^+^ calvaria cells were plated on 35 mm culture dishes at 0.5–1.0 × 10^4^ cells/cm^2^ with α‐MEM containing 10% FBS, 50 μg/mL ascorbic acid and antibiotics (osteogenic medium). Cells were treated with 10 mM β‐glycerophosphate for 2 days before culture termination and fixed in 4% paraformaldehyde in PBS for 10 minutes at 4°C. ALP and von Kossa staining were performed to determine mineralized nodules.^(^ ^22^ ^)^ All cultures were maintained at 37°C in a humidified atmosphere with 5% CO_2_, and medium was changed every second or third day.

### Fluorescence‐activated cell sorting (FACS)

Fractionated calvaria cells (fractions 3 to 6) were suspended in 250 μL of 2% FBS (PAA Laboratories GmbH, Pasching, Austria) in PBS (1–9 × 10^6^ cells/mL) and treated with 2.5 μL of DAPI (10 μg/mL) to exclude dead cells. After filtration (35 μm in pore size), cells were sorted on a BD FACSAria II flow cytometer (Franklin Lakes, NJ, USA) using a 130 μm nozzle at a flow rate of <3 on the flow rate scale from 1 to 11 (10–110 μL/min) to obtain Venus^+^ cell; these sorted cells were also histologically defined as Venus^+^ osteoblasts (see Results). Calvaria cells from wild‐type mice were used as a reference.

### Single‐cell RNA sequencing (RNA‐seq)

Isolated Venus^+^ osteoblasts (300 cells/μL) were loaded onto the C1 Single‐Cell mRNA Seq IFC (10‐ to 17‐μm cell diameter; Fluidigm, South San Francisco, CA, USA), and captured single cells were confirmed by phase‐contrast microscopy to exclude doublets and debris from further analysis. cDNAs were prepared in integrated fluidic circuits using the SMARTer Ultra Low RNA Kit for the Fluidigm C1 System (Takara Bio, Shiga, Japan). A bulk control (about 100 to 200 cells) and a negative (no template) control were processed in parallel using the same reagents and methods. Sequencing libraries were constructed in 96‐well plates using the Nextera XT DNA Sample Preparation Kit (Illumina, San Diego, CA, USA), according to protocols supplied by Fluidigm. Two hundred eighty‐five single‐cell libraries and control libraries were successfully collected and sequenced by either 100‐bp paired‐end on the Illumina HiSeq 2500 or 150‐bp paired‐end on the NovaSeq 6000. Quality metrics of single‐cell RNA‐seq data (except two samples for which a very low number of reads were obtained) were as follows: mean reads per cell, 3.96 million reads/cell; percentage of reads mapped to the genome, average 80.43%; total genes detected, 16,408 genes; mean detected genes per cell, 3854.13 genes/cell. These sequence data were deposited in DDBJ Sequence Read Archive under the accession number DRA011310 and DRA011348.

### Analyses of RNA‐seq data

Alignment of reads to UCSC *Mus musculus* transcriptome (mm10) and the calculations of read counts and fragments per kilobase of exon per million mapped fragments (FPKM) were done using the BaseSpace RNA‐seq Alignment v1.1.1 (http://basespace.illumina.com). Two samples were omitted from further analysis because of the very low number of reads obtained. Clustering and differential expression analyses were performed using the Seurat R package v3.0.0.^(^ ^23^, ^24^ ^)^ The Pearson's correlation coefficient was calculated using the Cor function in R. Violin plots were generated with the ggplot2 package in R. Highly variable genes were identified by using M3Drop, an R package.^(^ ^25^ ^)^ We used the Monocle R package v2.10.0 to do pseudotime analysis.^(^ ^26^, ^27^, ^28^ ^)^ Gene expression changes along pseudotime were analyzed by using the branched expression analysis modeling (BEAM) function in Monocle. Weighted gene co‐expression network analysis (WGCNA) was performed using the WGCNA R package.^(^ ^29^ ^)^ Gene ontology (GO) analysis was performed by using the PANTHER classification system (http://pantherdb.org/), and characteristic GO terms in category “biological process” were extracted from parent terms of hierarchy sort.^(^ ^30^ ^)^ Protein–protein interaction (PPI) network analysis was performed using the Cytoscape version 3.7.1 with the stringApp.^(^ ^31^ ^)^

## Results

### Venus^+^ cells

**Fig 1:**
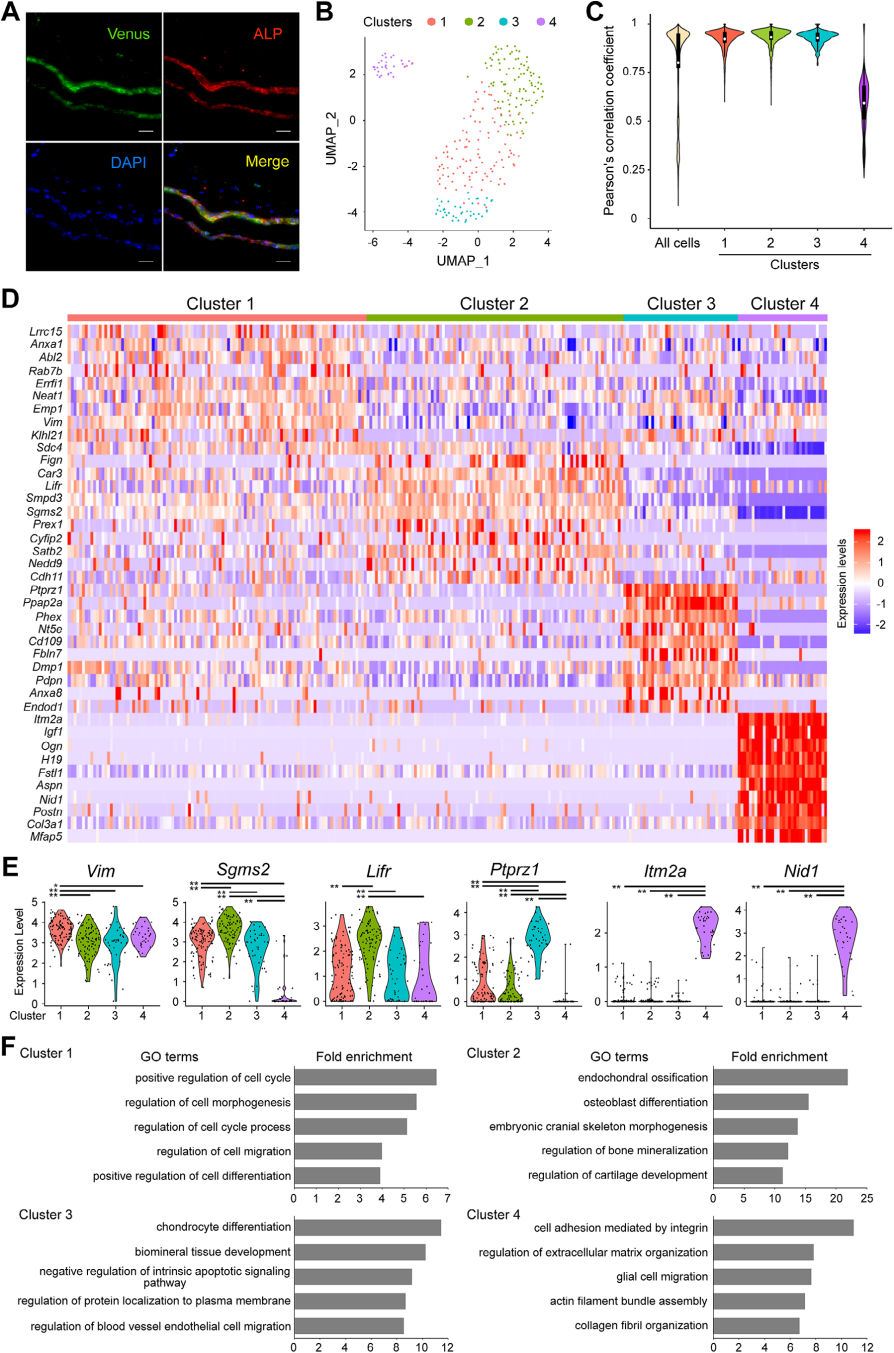
Clustering analysis of gene expression profiles of single Venus^+^ osteoblasts. (*A*) The distribution of Venus^+^ cells in calvariae of newborn *Col1a1‐Cre*; *R26R‐Lyn‐Venus* reporter mice. Paraffin‐embedded mouse calvariae were immunostained for Venus (green) and ALP (red). DAPI was used for nuclear counterstaining. Scale bars = 25 μm. (*B*) Venus^+^ osteoblast clusters by UMAP algorithm. Each dot denotes a single cell. Colors correspond to cell clusters. (*C*) The distribution of the Pearson's correlation coefficients of single‐cell transcriptomes. Data are shown as violin plots. (*D*) The top 10 marker genes in each cluster as determined by Seurat analysis. Genes and single cells are shown in rows and in columns of the heatmap, respectively. (*E*) The expression pattern of representative genes in each cluster. Dots denote single cells with violin plots. *p < 0.01, **p < 0.001. (*F*) The fold enrichment of the top 5 enriched GO terms (*p* < 0.05) in each cluster.

The distribution of cells expressing Venus in the calvariae of *Col1a1‐Cre*; *R26R‐Lyn‐Venus* newborn mice was confirmed by immunohistochemistry. ALP^+^ cells on bone surfaces, but not ALP^+^ cells further away from bone surfaces or ALP^+^ fibroblastic cells, were costained for Venus (Fig. 1*A*). In calvaria cell cultures fractionated by sequential enzymatic digestions, Venus^+^ cells were enriched in fractions 5 to 8 (Supplemental Fig. S1 *A*). Bone‐like mineralized nodules were found in fractions 2 to 8 cells cultured under osteogenic conditions but were much more abundant in fractions 5 to 8 versus earlier fractions (Supplemental Fig. S1 *B*). Venus^+^ cells were found exclusively in mineralized nodules and not surrounding cell layers (Supplemental Fig. S1 *C*). Based on these results, together with the elimination of osteocyte contamination by discarding fractions 7 and 8 (see Materials and Methods), we defined isolated fraction 3 to 6 Venus^+^ cells as osteoblasts (Venus^+^ osteoblasts).

### Clustering analysis of gene expression profiles of single Venus^+^ osteoblasts

We obtained the transcriptomes of 283 single Venus^+^ osteoblasts, and Seurat v3 was used to integrate the single‐cell data sets for characterization of Venus^+^ osteoblasts based on their gene expression profiles. Averaged single‐cell expression profiles were correlated with the corresponding bulk expression profiles (Supplemental Fig. S2 *A*). Uniform manifold approximation and projection (UMAP) and t‐distributed stochastic neighbor embedding (t‐SNE) analyses indicated that 272 Venus^+^ single osteoblasts could be divided into four clusters (cluster 1, 107 cells; cluster 2, 92 cells; cluster 3, 41 cells; cluster 4, 32 cells), and cluster 4 was completely isolated from clusters 1 to 3 (Fig. 1*B*, Supplemental Fig. S2 *B*). Eleven cells were classified as outlier cells. Cell‐to‐cell heterogeneity was quantified by Pearson's correlation coefficient and showed a broad spread (correlation coefficients *r* = 0.15 to 0.89, Fig. 1*C*), which was mostly attributed to cluster 4 (Fig. 1*C*). Cell‐to‐cell heterogeneity in gene expression was observed, with identification of 1516 highly variable genes across all cells (Supplemental Fig. S2 *C*, Supplemental Table S1). As shown in the Venn diagram (Supplemental Fig. S2 *D*), 1487 and 228 were observed as highly variable genes in clusters 1 to 3 and cluster 4 (Supplemental Tables S2 and S3), respectively. Comparison of gene expression profiles of one cluster with the others identified 1920 differentially expressed genes (*p* value <0.01, log fold‐change >0.25; Fig. 1 *D*, Supplemental Tables [Link], [Link]). We explored upregulated (more highly expressed) genes in one cluster versus the others to assign its cell‐type identity by measuring the receiver operating characteristic area under the curve (AUC; an AUC value of 1 represents a perfect classifier) and by assigning GO terms. In cluster 1, 94 genes were upregulated (Supplemental Table S4); the levels of these (see, for example, *Vim* with the highest AUC value 0.746, Fig. 1*E*) were slightly lower in other clusters. These genes were associated with the regulation of cell cycle, cell morphogenesis, cell migration, and cell differentiation (Fig. 1*F*). Cluster 2 showed 222 upregulated genes (Supplemental Table S5) including *Sgms2* (AUC = 0.803) and *Lifr* (AUC = 0.797) (Fig. 1*E*). This cluster was also characterized by unique enrichment of genes functionally relevant to bone formation (Fig. 1*F*). Cluster 3 showed 249 upregulated genes (Supplemental Table S6) having biological functions such as chondrocyte differentiation, biomineral tissue development, and negative regulation of apoptotic signaling pathway (Fig. 1*F*). Of these, the *Ptprz1* (AUC = 0.967; Fig. 1*E*), *Ppap2a* (*Plpp1*; AUC = 0.967), and *Phex* (AUC = 0.952) genes were ranked in the top three AUC values. As indicated above, cluster 4 was completely isolated from clusters 1 to 3 (Fig. 1*B*, Supplemental S2 *B*), and we therefore focused particularly on both upregulated (722 genes) and downregulated genes (755 genes) in cluster 4 (Supplemental Table S7). The enrichment of GO terms in cluster 4 was represented by cell adhesion, extracellular matrix organization, glial cell migration, actin filament bundle assembly, and collagen fibril organization (Fig. 1*F*). The genes *Itm2a* and *Nid1* with top‐ranked AUC values (AUC = 1.0 and 0.993, respectively) were expressed exclusively in cluster 4 (Fig. 1*E*). On the other hand, genes relating to endoplasmic reticulum (ER) to Golgi transport, retrograde transport from Golgi to ER, and osteoblast differentiation were downregulated in cluster 4 (Supplemental Fig. S2 *E*). Indeed, well‐known osteoblast markers, such as *Ibsp*, *Bglap*, and *Bglap2*, were ranked as the most downregulated genes in cluster 4 (Supplemental Table S7). PPI network analysis of up‐ and downregulated genes in cluster 4 showed that the *Il6* and *Egfr* genes (Supplemental Fig. S2 *F*) and the *Ctnnb1* and *Mtor* genes (Supplemental Fig. S2 *G*) function as hubs in cluster 4 and clusters 1 to 3, respectively.

### Expression profiles of osteogenic marker genes

**Fig 2:**
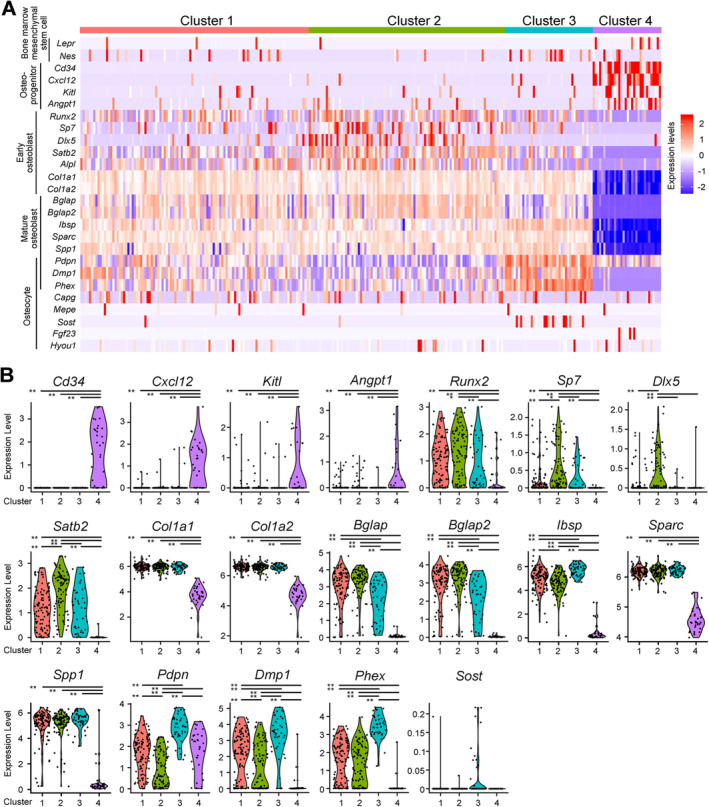
Identification of Venus^+^ osteoblast types based on known lineage marker genes. (*A*) The expression profiles of the selected osteoblast‐lineage marker genes. Data are shown as a heatmap. Genes and single cells are shown in rows and in columns of the heatmap, respectively. (*B*) The expression patterns of representative osteoblast‐lineage marker genes in each cluster. Dots denote single cells with violin plots. *p < 0.01, **p < 0.001.

A series of well‐established BMSC, osteoblast, and osteocyte marker genes were selected^(^ ^6^, ^7^, ^32^, ^33^, ^34^, ^35^, ^36^, ^37^, ^38^, ^39^ ^)^ to visualize their expression levels as a heatmap in the four clusters described above (Fig. 2*A*). This heatmap again showed clear distinction between cluster 4 and the others, suggesting that the expression profiles of established osteoblast marker genes may impinge on the clustering analysis. We then attempted to identify differentially expressed genes across each cluster (see details in Supplemental Tables [Link], [Link]). Consistent with cell fractionation based on Venus expression, the osteoblast marker genes *Col1a1*, *Col1a2*, *Sparc*, and *Spp1* were expressed in all single cells tested.^(^ ^18^, ^40^ ^)^ Notable, however, is that the expression levels of these genes were generally lower in cluster 4 versus clusters 1–3, whereas the *Cd34* and *Cxcl12* genes expressed in mesenchymal progenitor/osteoprogenitor cells^(^ ^32^, ^33^, ^35^, ^36^, ^37^ ^)^ were observed almost exclusively in cluster 4 (Fig. 2 *B*, Supplemental Tables [Link], [Link], and S13). Similarly, the hematopoietic stem cell niche factors *Kitl* and *Angpt1*, also known to be expressed in osteoprogenitor cells,^(^ ^39^ ^)^ were abundant in cluster 4 (Fig. 2*B*). Thus, cells in cluster 4 may represent a subset of osteoblasts that retain elements of mesenchymal progenitor/osteoprogenitor cell identities.

Although clusters 1–3 exhibit similar profiles of osteoblast lineage markers (Fig. 2*A*), a total of 909 genes were differentially expressed among these clusters (*p* < 0.01, log fold‐change >0.25; Supplemental Tables [Link], [Link], and S11), and several distinct features were evident (Fig. 2*B*). For example, the expression of the BMSC markers *Lepr* and *Nes* ^(^ ^38^ ^)^ was relatively rare in all clusters (Fig. 2*A*). The early osteoblast marker *Dlx5* was mostly found in cluster 2, which was further characterized by high levels of *Sp7* and *Satb2*. *Bglap* and *Bglap2*, mature osteoblast markers, showed lower expression in cluster 3 compared with clusters 1 and 2. The relative expression level of *Ibsp* was significantly different across clusters in the following order: cluster 3 > cluster 1 > cluster 2. *Pdpn*, *Dmp1*, and *Phex*, markers of the transition state between osteoblasts and osteocytes, were highest in cluster 3. Expression of other mature osteoblast markers, such as *Sparc* and *Spp1*, were not statistically different between clusters 1–3. With the exception of *Pdpn*, the osteoblast marker genes analyzed were less abundant in cluster 4 than in other clusters. Osteocyte marker genes were expressed in a few cells independently of cluster, with the exception of *Sost*, which exhibited an increasing expression trend in cluster 3 (Fig. 2*B*). Thus, cells in cluster 2 express a relatively less mature profile, followed by cells of cluster 1 and then cluster 3. We next generated a Venn diagram (Supplemental Fig. S3 *A*) and heatmap (Supplemental Fig. S3 *B*) from two lists of genes, ie, the highly variable genes (Supplemental Table S2) and the differentially expressed genes (Supplemental Tables [Link], [Link], and S11). This indicated that a total of 1300 genes were expressed independently of clusters 1 to 3. Overall, and consistent with previous studies,^(^ ^12^, ^13^ ^)^ these data suggest that Venus^+^ osteoblasts comprise cells that can be categorized into clusters representative of distinct stages of osteoblast differentiation, but that cells at such stages exhibit diverse gene expression profiles.

### Differentiation trajectory of osteoblasts

**Fig 3:**
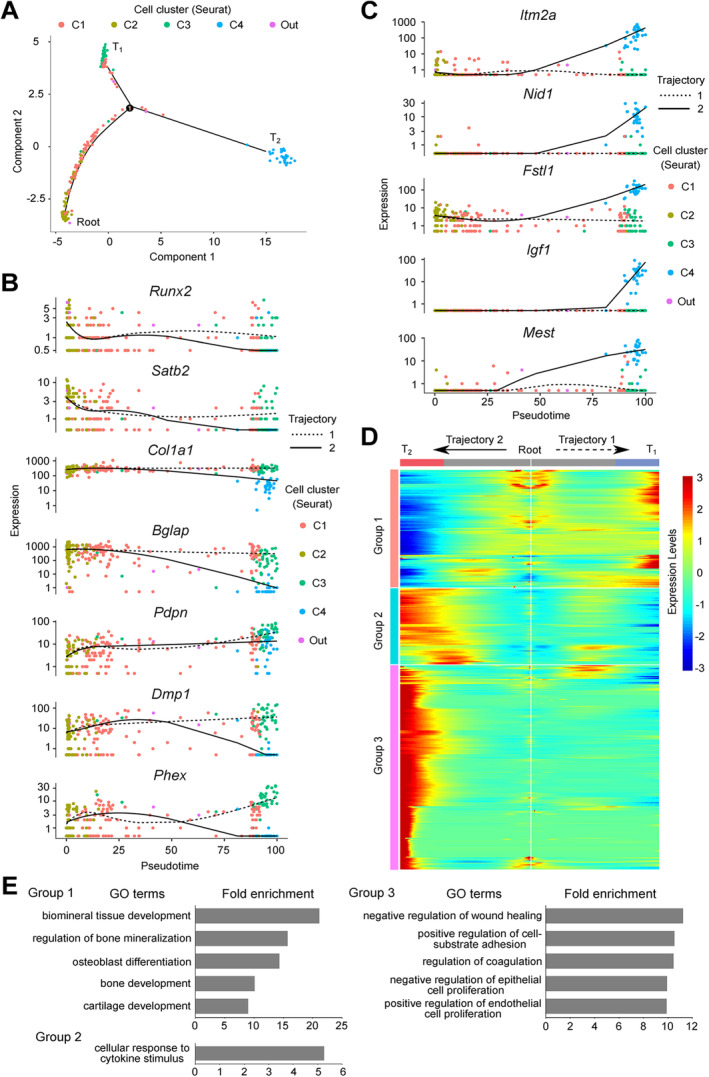
Pseudotemporal analysis of single Venus^+^ osteoblasts. (*A*) Pseudotemporal ordering of osteoblasts showing a root and developmental trajectory with a single bifurcation point splitting into two different terminals. Each dot corresponds to one single cell, colored according to its cluster label. Eleven cells labeled “Out” are outlier cells by Seurat analysis. (*B*) Representative osteoblast‐osteocyte gene expression kinetics along the pseudotime trajectories, from root to T_1_ (trajectory 1, dotted line) and to T_2_ (trajectory 2, solid line). Each dot corresponds to one single cell, colored according to its cluster label. (*C*) The top five ranked genes in AUC values of cluster 4 depict the expression kinetics along pseudotime trajectories from root to T_1_ (trajectory 1, dotted line) and T_2_ (trajectory 2, solid line). Each dot corresponds to one single cell, colored according to its cluster label. (*D*) The trajectory‐specific expression dynamics from root to T_1_ and T_2_. Genes (row) are clustered into three groups according to expression profiles and cells (column) are ordered according to the pseudotime. (*E*) The fold enrichment of the top 5 enriched GO terms (*p* < 0.05) in each group.

To further characterize the four clusters identified and address the osteoblast differentiation trajectory, we conducted pseudotime analysis using Monocle and ordered Venus^+^ osteoblasts in pseudotime. As shown in Fig. 3*A*, the pseudotime analysis arranged cells with a single bifurcation event giving rise to two distinct termini (denoted “terminal 1 (T_1_)” and “T_2_”). The cells at the root were composed of cells primarily belonging to cluster 2, with bifurcation toward either T_1_ (comprising mainly cluster 3 cells) or T_2_ (comprising cluster 4 cells). Cluster 1 cells were broadly distributed from root to T_1_, suggesting a linear trajectory from root to T_1_ (denoted “trajectory 1”), ie, sequential development within a restricted time window. On the other hand, interpretation of the linear trajectory from root to T_2_ (denoted “trajectory 2”) is less clear.

We therefore next characterized trajectories 1 and 2 by examining the expression kinetics of osteoblast marker genes along the pseudotime‐delineated trajectories (Fig. 3*B*). Trajectory 1 was characterized by the steep downregulation, then steady‐state expression of the early markers, *Runx2* and *Satb2*. *Col1a1* and *Bglap* expression remained constant through pseudotime. The transition state markers *Pdpn, Dmp1*, and *Phex* exhibited rapid early increases, slightly diminished expression (*Pdpn* and *Phex*), and then highest expression late. While the profiles of all these markers in trajectory 2 cells paralleled those of trajectory 1 at early times, all decreased to levels lower, in some cases (*Bglap*, *Dmp1*, *Phex*) much lower, than levels in trajectory 1. Together, these data support the view that trajectory 1 delineates cells during a limited time window of osteoblast development^(^ ^7^ ^)^ but that trajectory 2 delineates a distinctly different event/differentiation status. We also examined the expression kinetics of *Itm2a*, *Nid1*, *Fstl1*, *Igf1*, and *Mest* genes that ranked in the top five AUC values of cluster 4 (Supplemental Table S7). While the genes manifested steady‐state expression in trajectory 1, they were upregulated markedly in trajectory 2 (Fig. 3*C*).

We then used BEAM in Monocle to extract genes that were expressed in a trajectory‐dependent manner. A total of 403 genes were identified (*q* < 0.01; Supplemental Table S14) and their expression profiles visualized in a heatmap (Fig. 3*D*). According to expression similarities, these genes were classified into three groups: those with gradual increases and decreases in trajectory 1 and trajectory 2, respectively (group 1), those with transient and gradual increases in trajectory 1 and trajectory 2, respectively (group 2), and those exhibiting no changes and gradual increases in trajectory 1 and trajectory 2, respectively (group 3). GO analysis of genes in group 1 showed enrichment for terms related to bone formation (Fig. 3*E*), supporting the results obtained in pseudotime analysis. Trajectory 2 cells were again distinctly different, with enrichment in terms for functions including cellular response to cytokine stimulus, negative regulation of wound healing, positive regulation of cell‐substrate adhesion, regulation of coagulation, negative regulation of epithelial cell proliferation, and positive regulation of endothelial cell proliferation (Fig. 3*E*). Taken together, the data suggest that trajectory 1 delineates a differentiation process with continuous transition of osteoblasts to osteocytes, whereas trajectory 2 delineates cells apparently undergoing a reversal of osteoblast differentiation with acquisition of altered gene expression and potentially new function(s).

### Network analysis of single osteoblast transcriptomes

**Fig 4:**
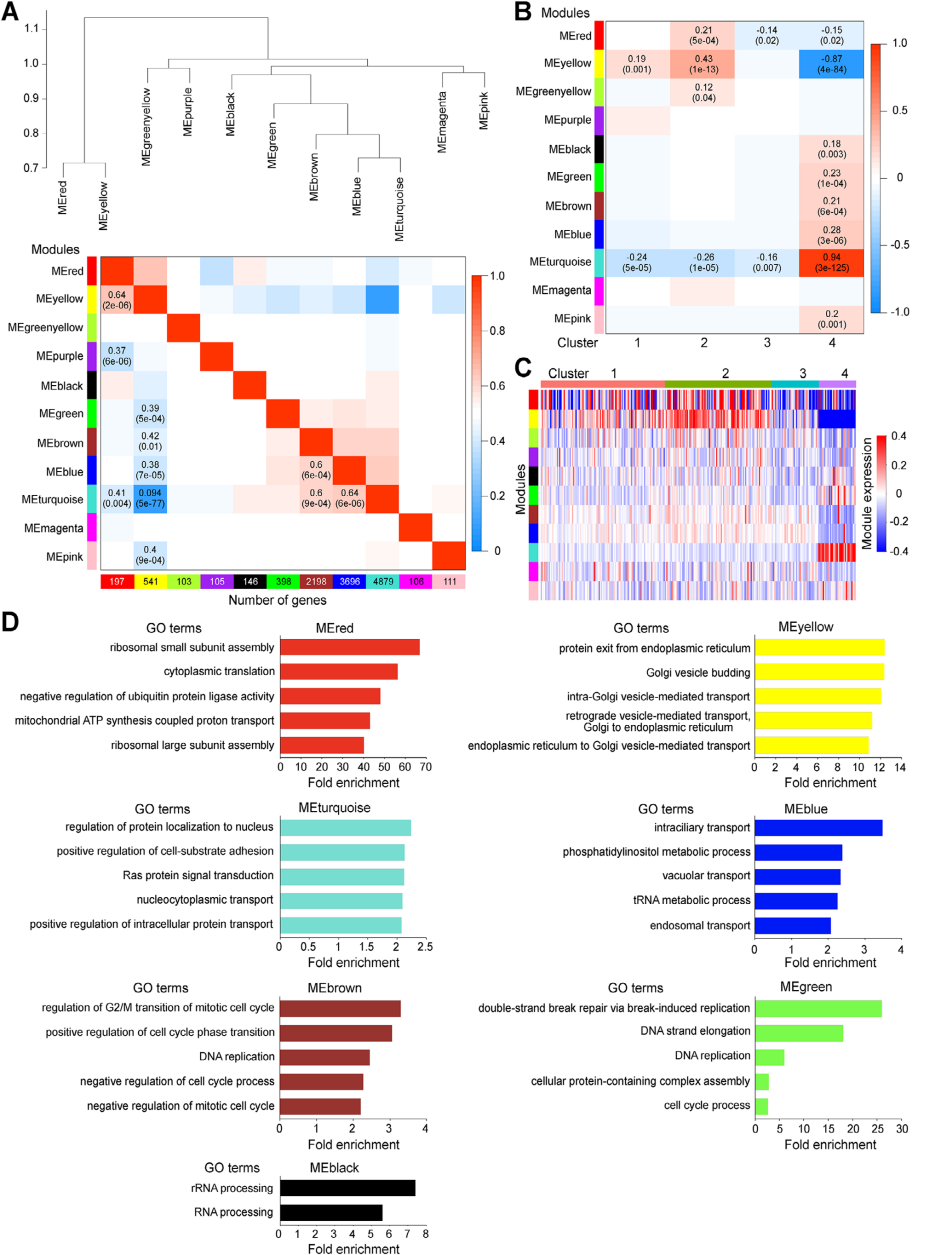
Network analysis of Venus^+^ osteoblast transcriptomes. (*A*) Upper panel: the hierarchical clustering dendrogram of module eigengenes; lower panel, module eigengene adjacency heatmap. Values (*p* < 0.01) in the heatmap indicate the degree of correlation between each module. The color scale indicates the correlation coefficient (blue = negative correlation; red = positive correlation). The *p* value is shown in parentheses. (*B*) Heatmap of the correlation between modules and clusters. The color scale and degrees (*p* values) are described in (*A*). (*C*) The average expression of module eigengenes. Modules and single cells in the heatmap are shown in rows and in columns, respectively. (*D*) The fold enrichment of the top 5 enriched GO terms (*p* < 0.05) in each module.

WGCNA was performed to construct a co‐expression network, which distinguished 11 distinct co‐expression module eigengenes. The eigengene dendrogram and the eigengene adjacency heatmap identified modules with high positive correlations that could be divided into two groups: the red and yellow modules and the others including turquoise, blue, and brown modules (Fig. 4*A*). These two groups showed a strong negative correlation (Fig. 4*A*), suggesting that the balanced expression of these module eigengenes may be significant for Venus^+^ osteoblasts. The red and yellow modules showed the highest correlation in cluster 2, followed by cluster 1 and cluster 3, and lowest correlation in cluster 4 (Fig. 4*B*), in accordance with their expression levels (Fig. 4*C*). GO analysis suggested that the red and yellow modules were associated with translation, ER to Golgi transport, and Golgi to ER retrograde transport (Fig. 4*D*). The green‐yellow module also showed a positive correlation with cluster 2 (Fig. 4*B*), but no enriched GO terms. It was, however, noted that the turquoise, blue, and brown modules showed positive correlation with cluster 4 (Fig. 4*B*). Of these, the turquoise module with abundant expression in cluster 4 (Fig. 4*C*) was enriched for genes involved in intracellular transport, cell‐substrate adhesion, and Ras protein signal transduction (Fig. 4*D*). GO analysis of all other modules significantly correlated with cluster 4 (except pink module with no enriched GO terms), including blue and brown modules, are also shown in Fig. 4*D*. Thus, cluster 4 cells exhibit a greater number of co‐expression modules than do cells of clusters 1 to 3, suggesting that the cells in cluster 4 are in a state of potential multifunctionality, while the cells in clusters 1 to 3 are more functionally uniform with focus on protein synthesis and transport.

## Discussion

Heterogeneity in the mRNA and protein repertoire expressed by osteoblasts was first documented more than 20 years ago by polyA‐PCR and immunocytochemistry analyses of single osteoblasts isolated from mineralizing colonies of cultured rat calvaria cells^(^ ^12^ ^)^ and by in situ hybridization and immunohistochemistry on sections of fetal rat calvariae.^(^ ^13^ ^)^ Evidence is now growing that the complex heterogeneous phenotype of a variety of cell types is biologically significant^(^ ^15^, ^41^, ^42^, ^43^, ^44^, ^45^, ^46^ ^)^ and that stochastic fluctuations in expression levels of genes and/or proteins drive cell fate determination.^(^ ^43^, ^44^ ^)^ It is, therefore, plausible that specific subpopulations of osteoblasts defined by differences in gene and/or protein expression are committed to different fates, ie, to apoptosis or to becoming osteocytes or bone lining cells.^(^ ^7^ ^)^ We have now extended data on single‐cell transcriptomes and employed a variety of machine learning tools to demonstrate the transcriptional heterogeneity of osteoblasts. To this end, we used a 2.3‐kb *Col1a1* promoter, which is activated concomitantly with *Ibsp* expression in vivo^(^ ^18^, ^40^ ^)^ and within a few days after endogenous *Col1a1* expression in mouse osteogenic cultures,^(^ ^47^ ^)^ to drive Venus expression in osteoblasts. These Venus^+^ osteoblasts were isolated from neonatal mouse calvariae. Our analysis, thus, was based on a limited osteoblast population and the number of analyzed cells was not large. Nevertheless, based on expression and machine learning analyses of the transcriptomes of 272 single Venus^+^ osteoblasts, we uncovered much more extensive heterogeneity of osteoblasts than previously documented, including several well‐established osteoblast differentiation markers^(^ ^6^, ^7^, ^32^, ^33^, ^34^, ^35^, ^36^, ^37^, ^38^, ^39^ ^)^ (see also below).

Dimension reduction methods (UMAP and t‐SNE analyses) indicated that the 272 Venus^+^ osteoblasts could be classified into four clusters. Seurat analysis revealed that a total of 1920 genes were differentially expressed across the clusters. GO analysis showed that cluster 1 was characterized by genes associated with the regulation of cell cycle and cell morphogenesis, cluster 2 with genes related to bone formation, cluster 3 with genes related to bone formation, apoptosis, and protein localization, and cluster 4 with genes involved in such activities as cell adhesion, extracellular matrix organization, and cell migration (Fig. 1*F*). Pseudotime ordering of the transcriptomes, including established osteoblast‐osteocyte markers, uncovered a developmental trajectory with root including cluster 2 (less mature osteoblasts), linear dispersion of cluster 1 (mature osteoblasts), and two distinct termini, cluster 3 (more mature osteoblasts) and cluster 4. In other words, while trajectory 1 delineated a sequence in which cluster 2 cells led to cluster 3 via cluster 1, ie, osteocytogenesis,^(^ ^7^ ^)^ trajectory 2 delineated a sequence in which mature osteoblasts (cluster 1) cells ended in cluster 4, ie, a distinctly different event/developmental status. In trajectory 1 (clusters 1 to 3), a total of 909 genes including established osteoblast markers, such as *Bglap*, *Ibsp*, and *Dmp1*, showed differential expression (Fig. 2*B*). Further, cells in cluster 1 were linearly dispersed from root to terminal 1 (T_1_), while cells in clusters 2 and 3 congregated mostly at root and T_1_, respectively. These results suggest that osteoblasts between root and T_1_ may cross a restricted time window of osteoblast development with markedly diverse gene expression profiles.

Unexpected was trajectory 2, ie, mature osteoblasts ending in cluster 4 with high expression of *Cd34* and *Cxcl12*, markers usually associated with less mature cells. For example, osteoprogenitor cells are enriched in the CD34^+^ population isolated from human and mouse bone marrow.^(^ ^32^, ^33^, ^37^ ^)^ Human CD34^+^ stromal cells can differentiate into fibroblasts, adipocytes, smooth muscle cells, and macrophages under appropriate conditions in long‐term culture.^(^ ^32^ ^)^ Concomitantly, CD34 levels are downregulated during osteogenic differentiation in mouse BMSC cultures.^(^ ^37^ ^)^ Likewise, CAR (CXC chemokine ligand 12, a transcriptional product of *Cxcl12*, expressing abundant reticular) cells have been characterized as mesenchymal progenitor cells, and osteoblasts fail to express *Cxcl12*.^(^ ^35^ ^)^ Given that both *Bglap‐Cre* and *Dmp1‐Cre* have been shown to target not only osteoblasts and osteocytes but also CAR cells,^(^ ^48^ ^)^ it is possible that CAR cells may also be a target of the 2.3‐kb *Col1a1* promoter. Taken together with our findings that single Venus^+^ osteoblasts expressed mature osteoblast marker genes, we conclude that cluster 4 cells are a unique subpopulation of osteoblasts that may retain or can reacquire progenitor properties. In this regard, a recent study has shown that bone lining cells express cell surface markers and genes characteristic of mesenchymal stem/progenitor cells, such as *Ly6a*, *Lepr*, and *Ctgf*, with coexpression of osteoblast markers *Dmp1* and *Phex*,^(^ ^8^ ^)^ in agreement with our data. Thus, cluster 4 cells may be a subpopulation of bone lining cells with broader mesenchymal progenitor cell characteristics. Recent single‐cell RNA‐seq analyses of stromal cells (bone marrow niche cells) isolated from mouse long bones suggest other possibilities.^(^ ^39^, ^49^ ^)^ Baryawno and colleagues suggest that there are two subsets of osteoblast lineage cells with distinct differentiation or lineage trajectories and with distinct hematopoietic support potential.^(^ ^39^ ^)^ Cluster 4 cells may have a different origin of differentiation because these cells expressed hematopoietic stem cell niche factors, such as *Cxcl12*, *Kitl1*, and *Angpt1* (Fig. 2*B*) and do not make a single continuous differentiation trajectory with clusters 1 to 3 in pseudotime analysis (Fig. 3*A*). A subpopulation of cells (referred to as *Fbn1* ^high^/*Igf1* ^high^ by Tikhonova and colleagues)^(^ ^49^ ^)^ also shows similar, but not identical, expression profiles to those of cluster 4 cells. If this subpopulation represents cells undergoing osteogenic transdifferentiation of chondrocytes to osteoblasts as Tikhonova and colleagues posit, our cluster 4 cells derived from calvariae (a tissue formed by intramembranous ossification) may be another subpopulation present only in certain tissues and/or at certain developmental stages. We also cannot exclude, however, the possibility that cluster 4 cells may be contaminated with some other type I collagen‐expressing mesenchymal progenitor cells, and further studies are clearly needed to characterize these cells. To this end, we attempted preliminary FACS fractionation experiments and found that the percentage of CD34^+^ cells in the Venus^+^ population was low (1.4%, data not shown), ie, lower than the estimated ~9% (vide supra, and Fig. 2) from our transcriptomic analyses. Further, not only the low yield of sorted Venus^+^ CD34^+^ cells but also their poor survival in culture (less than a week; data not shown) precluded our ability to characterize these cells more fully, an issue to be addressed in future.

As noted earlier, osteoblast fate includes not only conversion to osteocytes and lining cells but also apoptosis; however, we have not yet uncovered a well‐defined subpopulation of apoptotic cells in our analyses. Apoptosis is considered to be an essential component of various normal cellular processes, such as embryonic development, cell differentiation, and tissue homeostasis.^(^ ^50^ ^)^ Recent studies have shown that the conflicting signals of apoptosis and survival can be activated simultaneously through the same ligand‐receptor complex. Further, the magnitude of such signals not only varies among cell types but also depends on intrinsic and extrinsic noise even in the same cell type.^(^ ^50^ ^)^ Indeed, apoptotic response promotes osteoblast differentiation via the p53‐Akt‐FoxO pathway.^(^ ^51^ ^)^ Thus, expression of apoptotic versus survival signals and, concomitantly, apoptotic behavior may differ among individual osteoblasts, contributing to noise in the expression profiles and to our inability to recognize a distinct apoptotic subpopulation among the differentiating cells.

WGCNA analysis supports and extends the pseudotime differentiation trajectory analysis and the uniqueness of cluster 4 cells. WGCNA analysis showed that protein synthesis and protein traffic between ER and Golgi are active in root cells and decline through cells along trajectory 1 (Fig. 4). ER to Golgi trafficking is an essential prerequisite to sort and pack proteins for delivery to their final destinations, such as the extracellular space via secretory vesicles, plasma membrane, and other organelles,^(^ ^52^ ^)^ in keeping with the role of osteoblasts in extracellular matrix formation and mineralization. On the other hand, cluster 4 cells (the cells at the terminus of trajectory 2) showed greater expression of turquoise module eigengenes (Fig. 4), suggesting that cells in cluster 4 are active in intracellular transport, presumably transport proteins involved in signal transduction, such as Ras proteins (Fig. 4*D*). While Ras‐mediated signal transduction may be active in this cluster, phosphatidylinositol 3‐kinase signaling involved in phosphatidylinositol metabolism may not be functional because of the low levels of expression of blue module eigengenes. That the cells in this cluster may also be in a different phase of the cell cycle is suggested by the relatively low expression of brown module eigengenes, but this requires further studies given the relatively high expression of green module cell cycle–related genes (Fig. 4*C*, *D*).

Our data support the evolving concept of extensive biological diversity and developmental plasticity of osteoblasts with heterogeneous or distinct transcriptomes.^(^ ^34^, ^39^, ^49^ ^)^ Among this diversity, we identified a unique lineage‐committed osteoblastic cell type that expresses transcriptional features of progenitor cells (cluster 4). These cells may possess substantial cellular plasticity that allows dedifferentiation and reentry into the cell cycle to reprogram their cell fate, as observed previously, for example, in cardiomyocytes.^(^ ^53^ ^)^ In this regard, bone lining cells have been shown to display the ability to proliferate and contribute to bone formation after osteoblast ablation,^(^ ^8^ ^)^ suggesting that trajectory 2 may represent a process of dedifferentiation. Such transcriptional and biological diversity of osteoblasts may be achieved through cell‐to‐cell heterogeneity of epigenetic factors/mechanisms. Epigenetic heterogeneity has been suggested as a mechanistic component of fluctuating pluripotency in embryonic stem (ES) cells.^(^ ^54^ ^)^ How stochastic fluctuations in epigenetics and gene expression, even relatively small fluctuations often considered “noise,”^(^ ^43^, ^44^ ^)^ participate in these processes must be further explored. Nevertheless, we found approximately 1900 genes differentially expressed in four different osteoblast clusters that may offer potential new markers involved in osteocyte or lining cell fate determination. For example, *Sgms2*, a key regulator of sphingolipid signaling metabolites, and *Lifr*, a receptor for leukemia inhibitory factor, are known as causative genes for skeletal dysplasia^(^ ^55^, ^56^ ^)^ and Stüve‐Wiedemann/Schwartz‐Jampel type 2 syndrome,^(^ ^57^ ^)^ respectively. Thus, these genes may serve as markers of osteoblasts with divergent osteoblast activities (Fig. 1 *E*, Supplemental Table S5). LIFR is known to heterodimerize with gp130 to exert the inhibitory effect on osteoblastogenesis.^(^ ^58^ ^)^ The function of *Sgms2* in osteoblasts remains unclear, but it may be involved in the formation of osteoclasts.^(^ ^55^, ^56^ ^)^ *Ptprz1*, a member of the receptor tyrosine phosphatase family, and *Ppap2a* (*Plpp1*), a member of the phosphatidic acid phosphatase family, may delineate a subpopulation of osteoblasts capable of osteocyte differentiation (Fig. 1 *F*, Supplemental Table S6) by controlling the amount of extracellular lysophosphatidic acid.^(^ ^59^, ^60^ ^)^

We have now performed the first transcriptomic analysis of osteoblasts derived from neonatal mice calvariae at the single‐cell level, establishing a much greater extent of osteoblast heterogeneity than previously known. We have also clarified gradual fluctuations in gene expression during the differentiation and/or maturation processes of osteoblasts with higher resolution and more detail by analyzing a limited cell population. Our findings support the validity of and need for additional single‐cell analyses to determine mechanisms underlying osteoblast fate determination and the functional diversity of osteoblasts.

## Disclosures

All authors state that they have no conflicts of interest.

## Author contributions

**Hirotaka Yoshioka:** Conceptualization; data curation; formal analysis; funding acquisition; investigation; project administration; supervision; validation; visualization; writing‐original draft; writing‐review & editing. **Saki Okita:** Data curation; formal analysis; funding acquisition; investigation; validation; visualization; writing‐original draft. **Masashi Nakano:** Data curation; formal analysis; investigation; validation; visualization. **Tomoko Minamizaki:** Investigation. **Asako Nobukiyo:** Investigation. **Yusuke Sotomaru:** Investigation. **Edith Bonnelye:** Supervision; writing‐review & editing. **Katsuyuki Kozai:** Supervision. **Kotaro Tanimoto:** Supervision. **Jane Aubin:** Supervision; writing‐review & editing. **Yuji Yoshiko:** Conceptualization; funding acquisition; investigation; project administration; supervision; writing‐review & editing.

## Supporting information


**Supplemental Fig. S1.** Characterization of Venus^+^ cells in *Col1a1‐Cre; R26R‐Lyn‐Venus* mouse calvariae. (*A*) The relative abundance of Venus^+^ cells in each fraction. Data are shown as mean ± SD (*n* = 3–7). (*B*) Representative macroscopic images of cells with ALP/von Kossa staining. Cells were cultured in osteogenic medium for 19 days. (*C*) Immunofluorescence detection of Venus^+^ osteoblasts. Note that Venus^+^ osteoblasts reside almost exclusively in or close to mineralized nodules (see phase contrast).

**Supplemental Fig. S2.** Characterization of gene expression profiles of single Venus^+^ osteoblasts. (*A*) Scatter plot showing the correlation between the averaged single‐cell expression values and the averaged bulk expression values (two technical replicates). (*B*) Visualization of Venus^+^ osteoblast clusters by t‐SNE algorithm. Each dot denotes a single cell. (*C*) The squared coefficient of variation (CV^2^) against the average normalized read counts across all cells. The red line and red dashed line denote the fitted noise model and 95% confidence interval, respectively. (*D*) The overlap among the highly variable genes identified across all the cells, clusters 1 to 3 and cluster 4. (*E*) The fold enrichment of the top 5 enriched GO terms (*p* < 0.05) in clusters 1 to 3. (*F*, *G*) The PPI network of upregulated (*F*) and downregulated (*G*) genes in cluster 4. Node size represents betweenness centrality (larger nodes are more central), node colors represent the degree of connection (brighter colors are more connected nodes), and edge width represents edge‐betweenness values (thicker lines are higher values).

**Supplemental Fig. S3.** Heterogeneity of Venus^+^ osteoblasts in gene expression. (*A*) The overlap among the highly variable and differentially expressed genes across clusters 1 to 3. (*B*) The expression profiles of 100 representative genes as shown by heatmap.

**Supplemental Table S1.** Highly Variable Genes Across All Single Venus‐Positive Osteoblasts

**Supplemental Table S2.** Highly Variable Genes Across Single Venus‐Positive Osteoblasts in Clusters 1 to 3

**Supplemental Table S3.** Highly Variable Genes Across Single Venus‐Positive Osteoblasts in Cluster 4

**Supplemental Table S4.** Differentially Expressed Genes of Cluster 1 Versus Clusters 2 to 4

**Supplemental Table S5.** Differentially Expressed Genes of Cluster 2 Versus Clusters 1, 3, and 4

**Supplemental Table S6.** Differentially Expressed Genes of Cluster 3 Versus Clusters 1, 2, and 4

**Supplemental Table S7.** Differentially Expressed Genes of Cluster 4 Versus Clusters 1–3

**Supplemental Table S8.** Differentially Expressed Genes Between Clusters 1 and 2

**Supplemental Table S9.** Differentially Expressed Genes Between Clusters 1 and 3

**Supplemental Table S10.** Differentially Expressed Genes Between Clusters 1 and 4

**Supplemental Table S11.** Differentially Expressed Genes Between Clusters 2 and 3

**Supplemental Table S12.** Differentially Expressed Genes Between Clusters 2 and 4

**Supplemental Table S13.** Differentially Expressed Genes Between Clusters 3 and 4

**Supplemental Table S14.** Genes Involved in Developmental Trajectories in Single Venus‐Positive Osteoblasts

## References

[1] Prockop DJ . Marrow stromal cells as stem cells for nonhematopoietic tissues. Science. 1997;276(5309):71‐74. 9082988 10.1126/science.276.5309.71

[2] Nombela‐Arrieta C , Ritz J , Silberstein LE . The elusive nature and function of mesenchymal stem cells. Nat Rev Mol Cell Biol. 2011;12(2):126‐131. 21253000 10.1038/nrm3049 PMC3346289

[3] Komori T , Yagi H , Nomura S , et al. Targeted disruption of Cbfa1 results in a complete lack of bone formation owing to maturational arrest of osteoblasts. Cell. 1997;89(5):755‐764. 9182763 10.1016/s0092-8674(00)80258-5

[4] Nishio Y , Dong Y , Paris M , O'Keefe RJ , Schwarz EM , Drissi H . Runx2‐mediated regulation of the zinc finger Osterix/Sp7 gene. Gene. 2006;372:62‐70. 16574347 10.1016/j.gene.2005.12.022

[5] Hojo H , Ohba S , He X , Lai LP , McMahon AP . Sp7/Osterix is restricted to bone‐forming vertebrates where it acts as a Dlx co‐factor in osteoblast specification. Dev Cell. 2016;37(3):238‐253. 27134141 10.1016/j.devcel.2016.04.002 PMC4964983

[6] Long F . Building strong bones: molecular regulation of the osteoblast lineage. Nat Rev Mol Cell Biol. 2012;13(1):27‐38. 10.1038/nrm3254 22189423

[7] Dallas SL , Prideaux M , Bonewald LF . The osteocyte: an endocrine cell … and more. Endocr Rev. 2013;34(5):658‐690. 23612223 10.1210/er.2012-1026 PMC3785641

[8] Matic I , Matthews BG , Wang X , et al. Quiescent bone lining cells are a major source of osteoblasts during adulthood. Stem Cells. 2016;34(12):2930‐2942. 27507737 10.1002/stem.2474 PMC5450652

[9] Kim SW , Pajevic PD , Selig M , et al. Intermittent parathyroid hormone administration converts quiescent lining cells to active osteoblasts. J Bone Miner Res. 2012;27(10):2075‐2084. 22623172 10.1002/jbmr.1665 PMC3529414

[10] Yoshiko Y , Oizumi K , Hasegawa T , et al. A subset of osteoblasts expressing high endogenous levels of PPARγ switches fate to adipocytes in the rat calvaria cell culture model. PLoS One. 2010;5(7):e11782. 20668686 10.1371/journal.pone.0011782 PMC2909914

[11] Song L , Liu M , Ono N , Bringhurst FR , Kronenberg HM , Guo J . Loss of Wnt/β‐catenin signaling causes cell fate shift of preosteoblasts from osteoblasts to adipocytes. J Bone Miner Res. 2012;27(11):2344‐2358. 22729939 10.1002/jbmr.1694 PMC3474875

[12] Liu F , Malaval L , Aubin JE . The mature osteoblast phenotype is characterized by extensive plasticity. Exp Cell Res. 1997;232(1):97‐105. 9141626 10.1006/excr.1997.3501

[13] Candeliere GA , Liu F , Aubin JE . Individual osteoblasts in the developing calvaria express different gene repertoires. Bone. 2001;28(4):351‐361. 11336915 10.1016/s8756-3282(01)00410-0

[14] Kaneko K . Evolution of robustness to noise and mutation in gene expression dynamics. PLoS One. 2007;2(5):e434. 17502916 10.1371/journal.pone.0000434 PMC1855988

[15] Huang S . Non‐genetic heterogeneity of cells in development: more than just noise. Development. 2009;136(23):3853‐3862. 19906852 10.1242/dev.035139 PMC2778736

[16] Shapiro E , Biezuner T , Linnarsson S . Single‐cell sequencing‐based technologies will revolutionize whole‐organism science. Nat Rev Genet. 2013;14(9):618‐630. 23897237 10.1038/nrg3542

[17] Stegle O , Teichmann SA , Marioni JC . Computational and analytical challenges in single‐cell transcriptomics. Nat Rev Genet. 2015;16(3):133‐145. 25628217 10.1038/nrg3833

[18] Dacquin R , Starbuck M , Schinke T , Karsenty G . Mouse α1(I)‐collagen promoter is the best known promoter to drive efficient Cre recombinase expression in osteoblast. Dev Dyn. 2002;224(2):245‐251. 12112477 10.1002/dvdy.10100

[19] Abe T , Kiyonari H , Shioi G , et al. Establishment of conditional reporter mouse lines at ROSA26 locus for live cell imaging. Genesis. 2011;49(7):579‐590. 21445964 10.1002/dvg.20753

[20] Spatz JM , Wein MN , Gooi JH , et al. The Wnt inhibitor sclerostin is up‐regulated by mechanical unloading in osteocytes *in vitro* . J Biol Chem. 2015;290(27):16744‐16758. 25953900 10.1074/jbc.M114.628313 PMC4505423

[21] Xiong J , Piemontese M , Onal M , et al. Osteocytes, not osteoblasts or lining cells, are the main source of the RANKL required for osteoclast formation in remodeling bone. PLoS One. 2015;10(9):e0138189. 26393791 10.1371/journal.pone.0138189 PMC4578942

[22] Yoshiko Y , Wang H , Minamizaki T , et al. Mineralized tissue cells are a principal source of FGF23. Bone. 2007;40(6):1565‐1573. 17350357 10.1016/j.bone.2007.01.017

[23] Butler A , Hoffman P , Smibert P , Papalexi E , Satija R . Integrating single‐cell transcriptomic data across different conditions, technologies, and species. Nat Biotechnol. 2018;36(5):411‐420. 29608179 10.1038/nbt.4096 PMC6700744

[24] Stuart T , Butler A , Hoffman P , et al. Comprehensive integration of single‐cell data. Cell. 2019;177(7):1888‐902.e21. 31178118 10.1016/j.cell.2019.05.031 PMC6687398

[25] Andrews TS , Hemberg M . M3Drop: dropout‐based feature selection for scRNASeq. Bioinformatics. 2019;35(16):2865‐2867. 30590489 10.1093/bioinformatics/bty1044 PMC6691329

[26] Trapnell C , Cacchiarelli D , Grimsby J , et al. The dynamics and regulators of cell fate decisions are revealed by pseudotemporal ordering of single cells. Nat Biotechnol. 2014;32(4):381‐386. 24658644 10.1038/nbt.2859 PMC4122333

[27] Qiu X , Hill A , Packer J , Lin D , Ma Y‐A , Trapnell C . Single‐cell mRNA quantification and differential analysis with census. Nat Methods. 2017;14(3):309‐315. 28114287 10.1038/nmeth.4150 PMC5330805

[28] Qiu X , Mao Q , Tang Y , et al. Reversed graph embedding resolves complex single‐cell trajectories. Nat Methods. 2017;14(10):979‐982. 28825705 10.1038/nmeth.4402 PMC5764547

[29] Langfelder P , Horvath S . WGCNA: an R package for weighted correlation network analysis. BMC Bioinformatics. 2008;9:559. 19114008 10.1186/1471-2105-9-559 PMC2631488

[30] Mi H , Muruganujan A , Ebert D , Huang X , Thomas PD . PANTHER version 14: more genomes, a new PANTHER GO‐slim and improvements in enrichment analysis tools. Nucleic Acids Res. 2019;47(D1):D419‐D426. 30407594 10.1093/nar/gky1038 PMC6323939

[31] Shannon P , Markiel A , Ozier O , et al. Cytoscape: a software environment for integrated models of biomolecular interaction networks. Genome Res. 2003;13(11):2498‐2504. 14597658 10.1101/gr.1239303 PMC403769

[32] Simmons PJ , Torok‐Storb B . CD34 expression by stromal precursors in normal human adult bone marrow. Blood. 1991;78(11):2848‐2853. 1720038

[33] Chen J‐L , Hunt P , McElvain M , Black T , Kaufman S , Choi ES‐H . Osteoblast precursor cells are found in CD34^+^ cells from human bone marrow. Stem Cells. 1997;15(5):368‐377. 9323800 10.1002/stem.150368

[34] Aubin JE . Regulation of osteoblast formation and function. Rev Endocr Metab Disord. 2001;2(1):81‐94. 11704982 10.1023/a:1010011209064

[35] Omatsu Y , Sugiyama T , Kohara H , et al. The essential functions of adipo‐osteogenic progenitors as the hematopoietic stem and progenitor cell niche. Immunity. 2010;33(3):387‐399. 20850355 10.1016/j.immuni.2010.08.017

[36] Greenbaum A , Hsu Y‐MS , Day RB , et al. CXCL12 in early mesenchymal progenitors is required for haematopoietic stem‐cell maintenance. Nature. 2013;495(7440):227‐230. 23434756 10.1038/nature11926 PMC3600148

[37] Abdallah BM , Al‐Shammary A , Skagen P , et al. CD34 defines an osteoprogenitor cell population in mouse bone marrow stromal cells. Stem Cell Res. 2015;15(3):449‐458. 26413784 10.1016/j.scr.2015.09.005

[38] Kfoury Y , Scadden DT . Mesenchymal cell contributions to the stem cell niche. Cell Stem Cell. 2015;16(3):239‐253. 25748931 10.1016/j.stem.2015.02.019

[39] Baryawno N , Przybylski D , Kowalczyk MS , et al. A cellular taxonomy of the bone marrow stroma in homeostasis and leukemia. Cell. 2019;177(7):1915‐1932.e16. 31130381 10.1016/j.cell.2019.04.040 PMC6570562

[40] Rossert J , Eberspaecher H , de Crombrugghe B . Separate cis‐acting DNA elements of the mouse pro‐α 1(I) collagen promoter direct expression of reporter genes to different type I collagen‐producing cells in transgenic mice. J Cell Biol. 1995;129(5):1421‐1432. 7775585 10.1083/jcb.129.5.1421 PMC2120462

[41] Chambers I , Silva J , Colby D , et al. Nanog safeguards pluripotency and mediates germline development. Nature. 2007;450(7173):1230‐1234. 18097409 10.1038/nature06403

[42] Dietrich J‐E , Hiiragi T . Stochastic patterning in the mouse pre‐implantation embryo. Development. 2007;134(23):4219‐4231. 17978007 10.1242/dev.003798

[43] Chang HH , Hemberg M , Barahona M , Ingber DE , Huang S . Transcriptome‐wide noise controls lineage choice in mammalian progenitor cells. Nature. 2008;453(7194):544‐547. 18497826 10.1038/nature06965 PMC5546414

[44] Raj A , van Oudenaarden A . Nature, nurture, or chance: stochastic gene expression and its consequences. Cell. 2008;135(2):216‐226. 18957198 10.1016/j.cell.2008.09.050 PMC3118044

[45] Kobayashi T , Mizuno H , Imayoshi I , Furusawa C , Shirahige K , Kageyama R . The cyclic gene Hes1 contributes to diverse differentiation responses of embryonic stem cells. Genes Dev. 2009;23(16):1870‐1875. 19684110 10.1101/gad.1823109 PMC2725939

[46] Spencer SL , Gaudet S , Albeck JG , Burke JM , Sorger PK . Non‐genetic origins of cell‐to‐cell variability in TRAIL‐induced apoptosis. Nature. 2009;459(7245):428‐432. 19363473 10.1038/nature08012 PMC2858974

[47] Dacic S , Kalajzic I , Visnjic D , Lichtler AC , Rowe DW . Col1a1‐driven transgenic markers of osteoblast lineage progression. J Bone Miner Res. 2001;16(7):1228‐1236. 11450698 10.1359/jbmr.2001.16.7.1228

[48] Zhang J , Link DC . Targeting of mesenchymal stromal cells by Cre‐recombinase transgenes commonly used to target osteoblast lineage cells. J Bone Miner Res. 2016;31(11):2001‐2007. 27237054 10.1002/jbmr.2877 PMC5523961

[49] Tikhonova AN , Dolgalev I , Hu H , et al. The bone marrow microenvironment at single‐cell resolution. Nature. 2019;569(7755):222‐228. 30971824 10.1038/s41586-019-1104-8 PMC6607432

[50] Flusberg DA , Sorger PK . Surviving apoptosis: life–death signaling in single cells. Trends Cell Biol. 2015;25(8):446‐458. 25920803 10.1016/j.tcb.2015.03.003 PMC4570028

[51] Komori T . Cell death in chondrocytes, osteoblasts, and osteocytes. Int J Mol Sci. 2016;17(12):2045. 10.3390/ijms17122045 PMC5187845 27929439

[52] Geva Y , Schuldiner M . The back and forth of cargo exit from the endoplasmic reticulum. Curr Biol. 2014;24(3):R130‐R136. 24502791 10.1016/j.cub.2013.12.008

[53] Zhang Y , Li T‐S , Lee S‐T , et al. Dedifferentiation and proliferation of mammalian cardiomyocytes. PLoS One. 2010;5(9):e12559. 20838637 10.1371/journal.pone.0012559 PMC2933247

[54] Angermueller C , Clark SJ , Lee HJ , et al. Parallel single‐cell sequencing links transcriptional and epigenetic heterogeneity. Nat Methods. 2016;13(3):229‐232. 26752769 10.1038/nmeth.3728 PMC4770512

[55] Pekkinen M , Terhal PA , Botto LD , et al. Osteoporosis and skeletal dysplasia caused by pathogenic variants in SGMS2. JCI Insight. 2019;4(7):e126180. 10.1172/jci.insight.126180 PMC6483641 30779713

[56] Robinson M‐E , Bardai G , Veilleux L‐N , Glorieux FH , Rauch F . Musculoskeletal phenotype in two unrelated individuals with a recurrent nonsense variant in SGMS2. Bone. 2020;134:115261. 32028018 10.1016/j.bone.2020.115261

[57] Dagoneau N , Scheffer D , Huber C , et al. Null leukemia inhibitory factor receptor (LIFR) mutations in Stüve‐Wiedemann/Schwartz‐Jampel type 2 syndrome. Am J Hum Genet. 2004;74(2):298‐305. 14740318 10.1086/381715 PMC1181927

[58] Malaval L , Liu F , Vernallis AB , Aubin JE . GP130/OSMR is the only LIF/IL‐6 family receptor complex to promote osteoblast differentiation of calvaria progenitors. J Cell Physiol. 2005;204(2):585‐593. 15751050 10.1002/jcp.20312

[59] Tomsig JL , Snyder AH , Berdyshev EV , et al. Lipid phosphate phosphohydrolase type 1 (LPP1) degrades extracellular lysophosphatidic acid *in vivo* . Biochem J. 2009;419(3):611‐618. 19215222 10.1042/BJ20081888 PMC2677185

[60] Wu X , Ma Y , Su N , Shen J , Zhang H , Wang H . Lysophosphatidic acid: its role in bone cell biology and potential for use in bone regeneration. Prostaglandins Other Lipid Mediat. 2019;143:106335. 31054330 10.1016/j.prostaglandins.2019.106335

