# Supplementary figures and images for: Single‐Cell RNA‐Sequencing Reveals the Breadth of Osteoblast Heterogeneity
Source: JBMR Plus. 2021 May 17;5(6):e10496. doi: 10.1002/jbm4.10496 (PMC8216137; doi:10.1002/jbm4.10496)

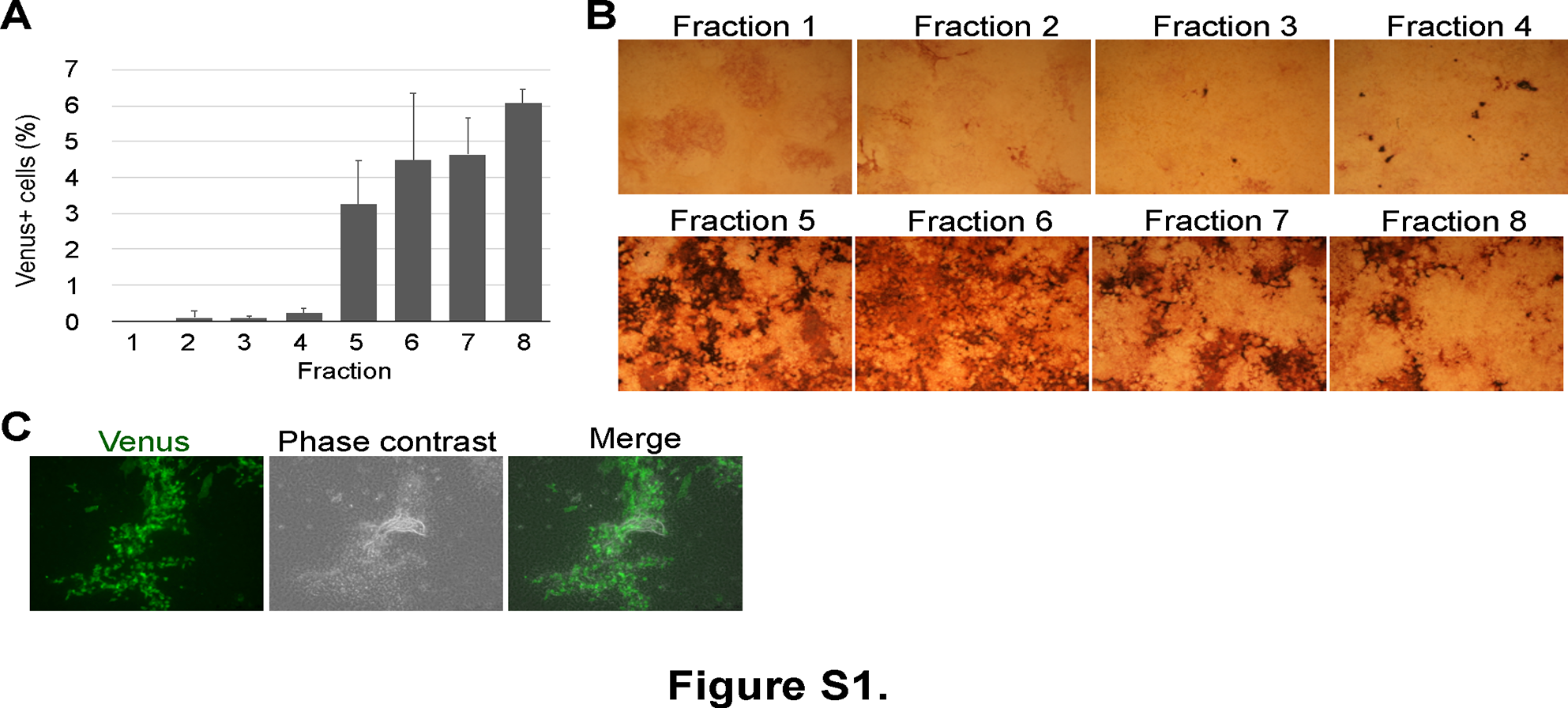

Supplement: Supplementary file 1 — Supplemental Fig. S1. Characterization of Venus+ cells in Col1a1‐Cre; R26R‐Lyn‐Venus mouse calvariae. (A) The relative abundance of Venus+ cells in each fraction. Data are shown as mean ± SD (n = 3–7). (B) Representative macroscopic images of cells with ALP/von Kossa staining. Cells were cultured in osteogenic medium for 19 days. (C) Immunofluorescence detection of Venus+ osteoblasts. Note that Venus+ osteoblasts reside almost exclusively in or close to mineralized nodules (see phase contrast). [file JBM4-5-e10496-s008.tiff]

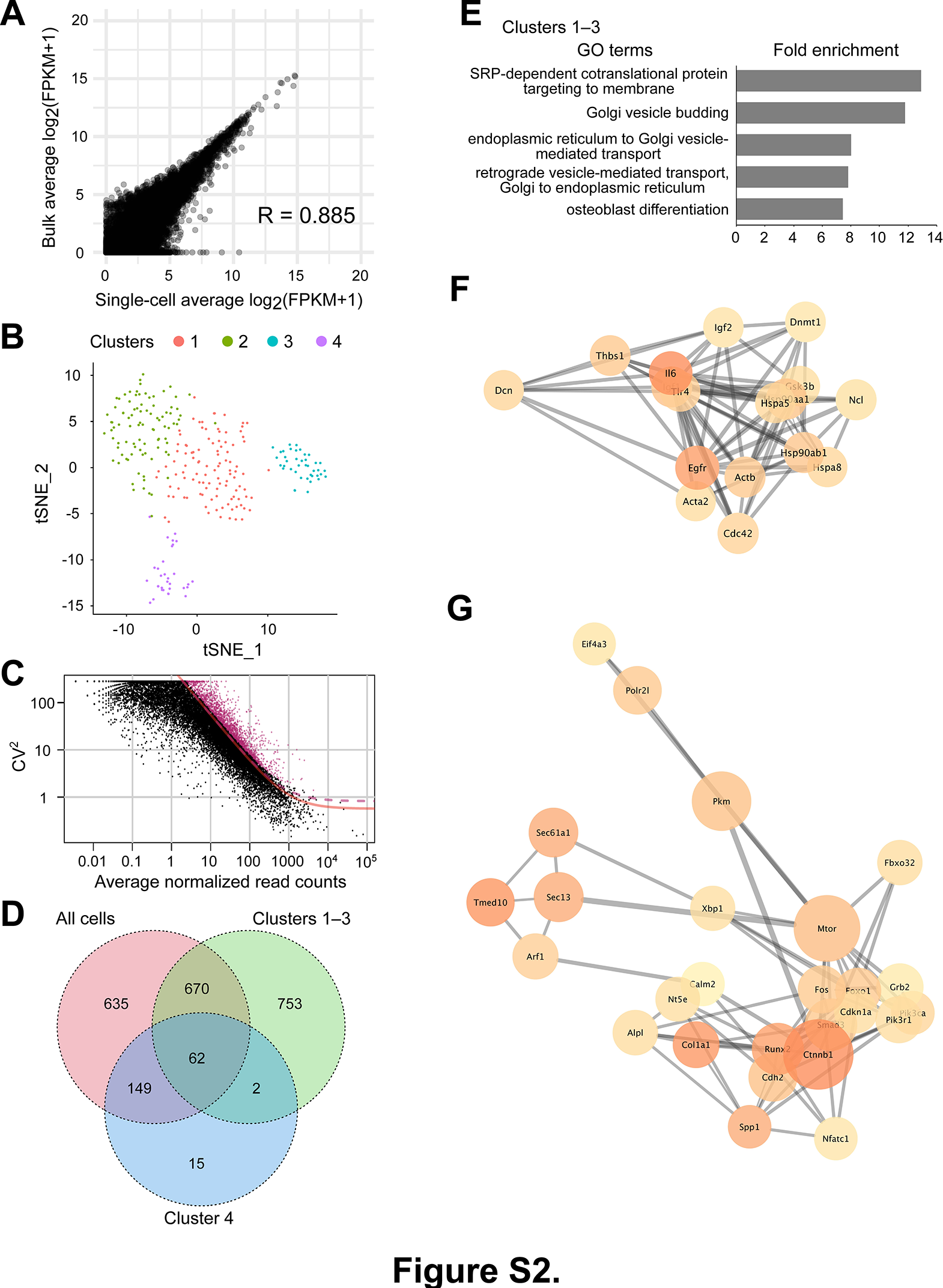

Supplement: Supplementary file 2 — Supplemental Fig. S2. Characterization of gene expression profiles of single Venus+ osteoblasts. (A) Scatter plot showing the correlation between the averaged single‐cell expression values and the averaged bulk expression values (two technical replicates). (B) Visualization of Venus+ osteoblast clusters by t‐SNE algorithm. Each dot denotes a single cell. (C) The squared coefficient of variation (CV2) against the average normalized read counts across all cells. The red line and red dashed line denote the fitted noise model and 95% confidence interval, respectively. (D) The overlap among the highly variable genes identified across all the cells, clusters 1 to 3 and cluster 4. (E) The fold enrichment of the top 5 enriched GO terms (p < 0.05) in clusters 1 to 3. (F, G) The PPI network of upregulated (F) and downregulated (G) genes in cluster 4. Node size represents betweenness centrality (larger nodes are more central), node colors represent the degree of connection (brighter colors are more connected nodes), and edge width represents edge‐betweenness values (thicker lines are higher values). [file JBM4-5-e10496-s013.tiff]

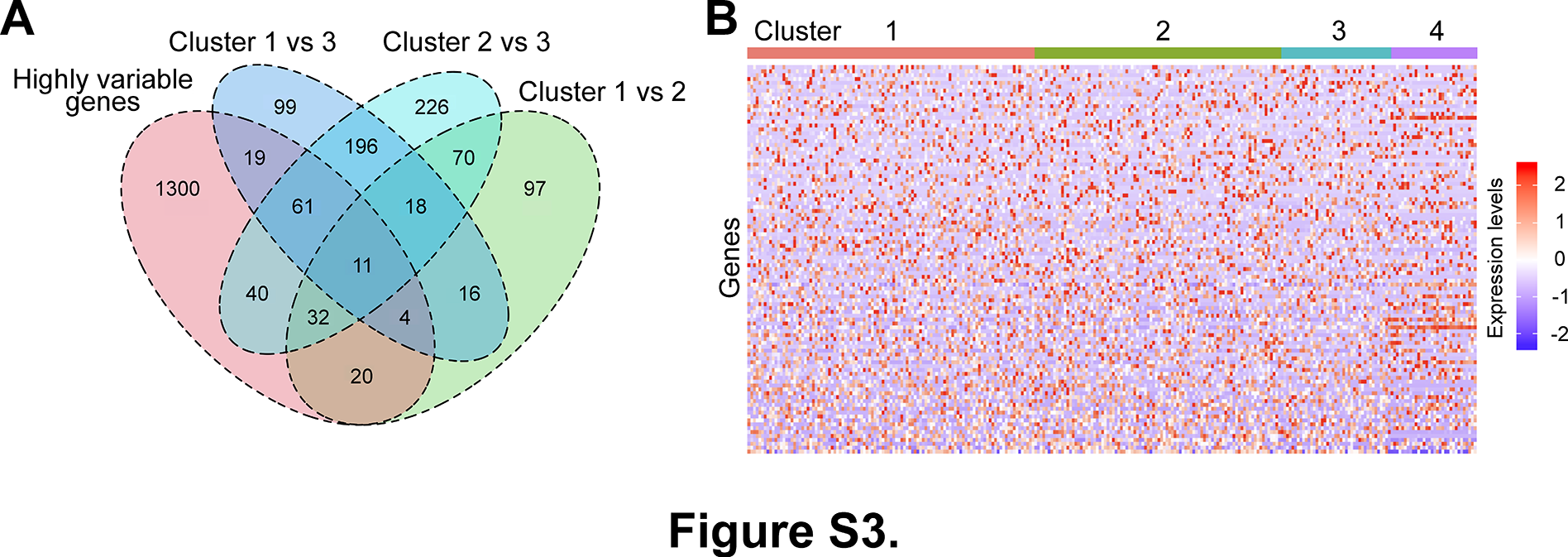

Supplement: Supplementary file 3 — Supplemental Fig. S3. Heterogeneity of Venus+ osteoblasts in gene expression. (A) The overlap among the highly variable and differentially expressed genes across clusters 1 to 3. (B) The expression profiles of 100 representative genes as shown by heatmap. [file JBM4-5-e10496-s005.tiff]
